# Comprehensive Analysis of Prognostic and immune infiltrates for FOXPs Transcription Factors in Human Breast Cancer

**DOI:** 10.1038/s41598-022-12954-3

**Published:** 2022-05-25

**Authors:** Jianing Yi, Siyi Tan, Yuanjun Zeng, Lianhong Zou, Jie Zeng, Chaojie Zhang, Luyao Liu, Peizhi Fan

**Affiliations:** 1 Surgical Department of Breast and Thyroid Gland, Hunan Provincial People’s Hospital, The First Affiliated Hospital of Hunan Normal University, Changsha, Hunan People’s Republic of China; 2 Surgical Department of Medical Laboratory, Huazhi Biotechnology Co. Ltd, Changsha, Hunan People’s Republic of China; 3 Department of Pathology, Hunan Provincial People’s Hospital, The First Affiliated Hospital of Hunan Normal University, Changsha, Hunan People’s Republic of China; 4 Institute of Translational Medicine, Hunan Provincial People’s Hospital, The First Affiliated Hospital of Hunan Normal University, Changsha, Hunan People’s Republic of China

**Keywords:** Breast cancer, Immunology

## Abstract

Forkhead-box-P family include FOXP1/2/3/4 and its clinical significance still remains unclear in breast cancer (BRCA). We analysed the expressions of FOXPs in BRCA patients to determine diagnostic and prognostic values. Our results indicated that the transcriptional levels of FOXP3/4 were up-regulated in BRCA patients, but FOXP2 were down-regulated. No statistically significant correlation were found between the expression levels of FOXPs in Pathologic stage. FOXP2/3 had a significantly high AUC value in the detection of breast cancer, with 96.8% or 95.7% in accuracy respectively. Our study also suggested that BRCA patients with high transcription levels of FOXP1/2/4 were significantly associated with longer Overall Survival (OS). In contrast, BRCA patients with high transcription level of FOXP3 was not statistically related with OS. Our work revealed that FOXPs were closely related to the alteration of extensive immune checkpoints in breast invasive carcinoma. Additionally, FOXP3 has a significant positive correlation with PDCD1, CD274, CTLA4 and TMB in breast cancer, and FOXP3 expression showed a statistically significant correlation with infiltration of immune cells. Finally, we found that FOXP3 expression predicted the breast cancer cells response to anticancer drugs. Altogether, our work strongly suggested that FOXPs could serve as a biomarker for tumor detection, therapeutic design and prognosis.

## Introduction

Surpassing lung cancer, female breast cancer has now been in the leading position of the world cancer incidence in 2020, with about 2.3 million new cases, accounting for 11.7% of all cancer cases. It is the fifth cause of cancer mortality in the world, with 685,000 deaths^1^.

Despite the recent advanced treatments made in breast cancer treatments include earlier detection, surgery, chemotherapy, immunotherapy and targetting drug therapy^2,3^. In 5–10% of the breast cancer patients, tumors have already expanded to the advanced stage with extensive lymph invasion or distant metastasis when diagnosed, there is still poor survival rate of these metastatic breast cancer patients, of these patients only one-fifth survive 5 years^4^.

Due to individual difference and tumor heterogeneity, the current diagnostic and prognostic biomarkers for breast cancer have some limitations^5^, It is therefore imperative demand to investigate more effective diagnostic and prognostic biomarkers for optimizing the management of breast cancer.

Cancer progression is involved in epigenetic and genetic alterations including transcription factors, growth factors, cytokines, and proteases under tumor microenvironment^6^. As one of transcription factors, Forkhead box P (FOXP) family include FOXP1, FOXP2, FOXP3 and FOXP4 with similar 110 amino acid DNA-binding domain termed forkhead domain^7^. FOXP proteins can regulate gene transcription in connection with carcinogenesis^8^, immune function^9^, invasion and metastasis of carcinoma^10^, differentiation^11^ and angiogenesis^12^.

Accumulating evidence shows that FOXP family proteins have dual biologic functions as an oncogene or a tumor suppressor. Diffuse large B-cell lymphoma with overexpressed FOXP1 has poor prognosis^13^, while FOXP1 acts as a tumor suppressor in breast and lung carcinoma^14,15^. CD4+/CD25+/FOXP3+ Treg cells work in gastric cancers through immunosuppression as oncogenes, while FOXP3 overexpression in patients with breast cancers indicates good prognosis as a tumor suppressor^16,17^.

It remains unclear that clinical significance of FOXP family proteins act as an entirety in human breast cancer^18^. Bioinformatics analysis has been applied to survey the role of transcription factors in breast cancer. In the present study, according to the analyses of gene expressions or variations in published online, we analysis the expressions and mutations of different FOXP factors in patients with breast cancer in detail to determine diagnostic and prognostic values of FOXP in breast cancer.

## Methods

### Oncomine database analysis

The expression level of the FOXPs in various types of cancers was identified in the Oncomine database (https://www.oncomine.org/resource/login.html)^19^. The threshold was determined according to the following values: *P* value of 0.01, fold change of 1.0, and gene ranking of all.

### Tumor immune estimation resource (TIMER) database

TIMER is a comprehensive online resource for systematic analysis of immune infiltrates across various cancer types^20^. In this study, we observed the expression difference of FOXPs between tumor and adjacent normal tissues for the BRCA of the TCGA project. Meanwhile, we performed TIMER to determine the relationship between FOXPs expression in BRCA and 6 immune infiltrates (B cells, CD4+ T cells, CD8+ T cells, neutrophils, macrophages and dendritic cells).

### RNA-sequencing data of FOXPs in human BRCA

The RNA-Seq expression data of FOXPs in BRCA was downloaded from TCGA (https://portal.gdc.cancer.gov/). Therefore, 113 adjacent normal tissues and 1109 BRCA data were retained. The samples selected contained FOXPs gene expression data and associated clinical information, including age, gender, Pathological stage, Race, Histological type.

### Immunohistochemistry

Clinical samples were obtained from breast cancer patients who were surgically treated at Hunan Provincial People’s Hospital/The First Affiliated Hospital of Hunan Normal University. Tumor tissue and its adjacent normal tissues were prepared into 4 mm paraffin sections and incubated with primary rabbit monoclonal antibodies of FOXP1, FOXP2, FOXP3, FOXP4 (1:200 dilution; Santa Cruz Biotechnology, Santa Cruz, USA) at 4° overnight. The sections were coupled with goat anti-rabbit antibody labeled with horseradish peroxidase (1:400, Abcam, USA) at room temperature for 60 min, then each section was stained with 3,3-diaminobenzidine (DAB) reagent, and finall weakly counterstained with hematoxylin.

### The Kaplan–Meier plotter analysis

The Kaplan–Meier plotter (www.kmplot.com)^21,22^ was used to assess the prognostic value of FOXPs mRNA expression in BRCA patients and analyzed the overall survival (OS), progression-free survival (PFS), post-progression survival (PPS) and distant metastasis-free survival (DMFS) of patients with BRCA. The patients divided into high expression groups and low expression groups according to the median values of FOXPs mRNA expression.

### The cBioPortal analysis

We selected a Breast Invasive Carcinoma dataset (TCGA, Firehose Legacy) that contained 1109 pathological reports to analyzed the expression of FOXPs and immune checkpoints using cBioPortal (www.cbioportal.org)^23,24^. The genomic map contains putative copy-number alterations (CNA) from GISTIC, mRNA expression z-scores and Protein expression z-scores mutations.

### STRINGS analysis

STRINGS (www.string-db.org) is an online tool for analysis of all publicly data of protein–protein interaction (PPI)^25^. In this study, we used a PPI network analysis on FOXPs to explore their functions in human breast cancer.

### GeneMANIA analysis

GeneMANIA (www.genemania.org) is an online tool for analysis of gene functions^26^. In this study, we performed GeneMANIA to select the 50 most important genes to construct gene–gene interaction network for FOXPs.

### Drug–gene interactions

DGidb^27^ (https://dgidb.genome.wustl.edu/), a web server for discovering drug–gene interactions or potentially available drug categories, was used to explore the potential druggable genes and drugs of FOXPs in patients.

### Statistical analyses

All statistical analyses were implemented with R (www.r-project.org). gene expression data and clinical information were visualizes by R package “ggplot2” R package^28^. ROC curve was performed to detect the cutoff value of FOXPs, BRCA1, BRCA2 and ERBB2. Lollipop chart used Spearman’s correlation analysis to describe the correlation between 24 immune cell^29,30^. TMB Score: We used Spearman’s correlation analysis to describe the correlation between quantitative variables without a normal distribution. A *P* value of less than 0.05 was considered statistically significant.

### Ethics statement

All experiments involving clinical samples (Informed consent was obtained from all subjects and/or their legal guardian(s)) were approved by the Ethics Committee of Hunan Provincial People’s Hospital/The First Affiliated Hospital of Hunan Normal University (document NO.202193). All the experiments were conducted in accordance with ethical guidelines, including the tenets of the Declaration of Helsinki.

### Significance

Our study strongly suggests that FOXPs could serve as a biomarker for tumor detection, therapeutic design and prognosis.

## Results

### Transcriptional levels of FOXPs in BRCA patients

**Figure 1:**
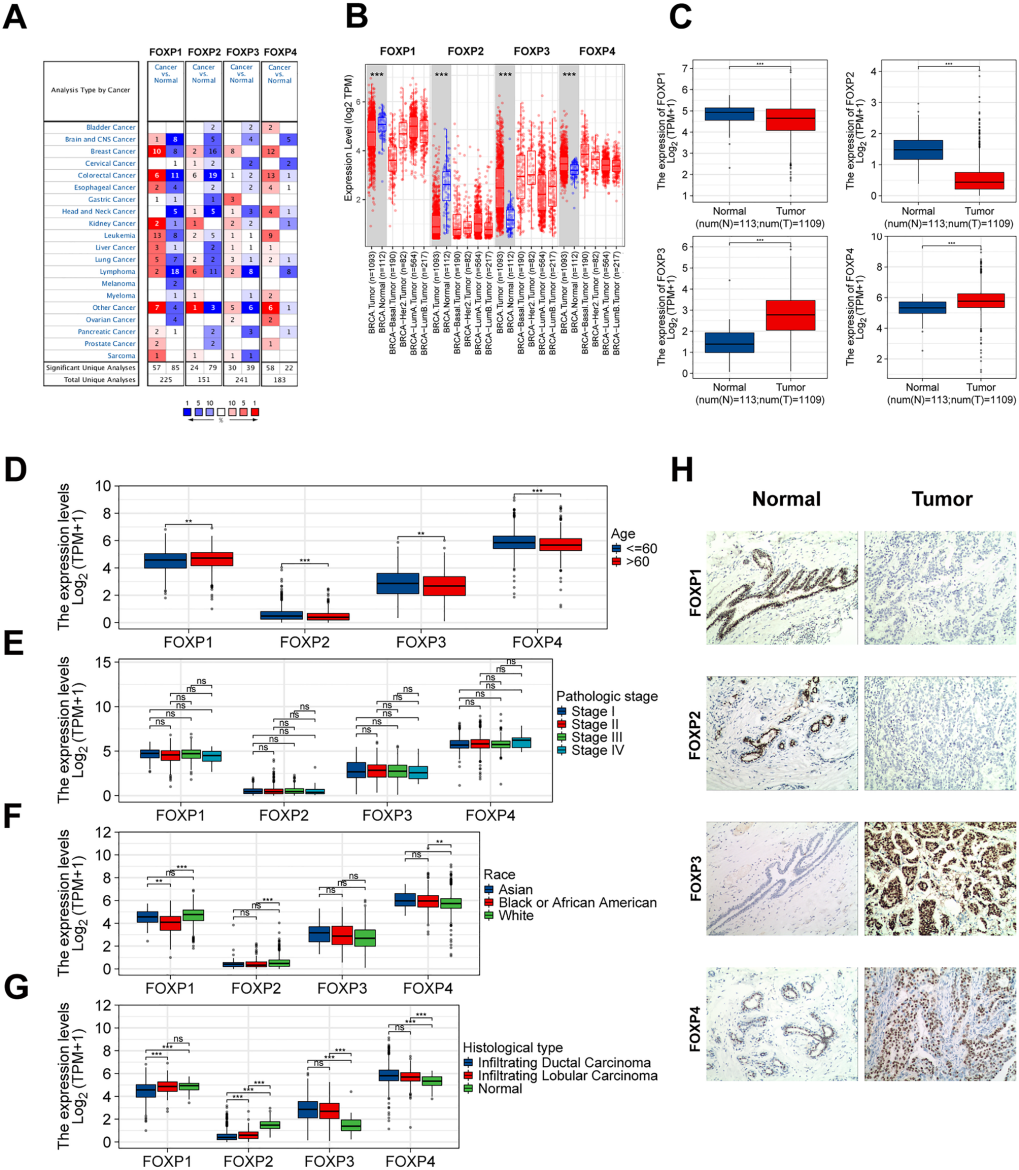
FOXPs expression levels, clinical characteristics and immunohistochemistry in human cancer. (**A**) The transcription levels of FOXPs in different types of cancers (Oncomine). (**B**) Human FOXPs expression levels in human BRCA from TCGA database were determined (TIMER). (**C**) The mRNA expression levels of FOXPs in 1109 BRCA samples and 113 normal samples from TCGA. (**D**) FOXP2,3,4 mRNA expression were significantly higher in patients (> = 60) than in patients (< 60), however the result was the opposite in FOXP1. (**E**) No statistically significant correlation were found between the expression levels of FOXPs in Pathologic stage. (**F**) The expression of FOXPs was different among different races. (**G**) FOXP1,2 mRNA expression were significantly higher in Infiltrating Lobular Carcinoma than Infiltrating Ductal Carcinoma, however no statistically significant correlation were found between FOXP3,4. (**H**) The Expression of FOXPs in BRCA (IHC) (ns, no significance, *P < 0.05, **P < 0.01, ***P < 0.001).

Four FOXPs are generally found in mammalian cells, but are expressed abnormally in different tumor tissues. We performed the Oncomine and TIME to compare the mRNA expression of FOXPs in different cancer and normal tissue samples (Fig. 1A,B). The results showed that the transcriptional levels of FOXP3/4 were up-regulated in BRCA patients, but FOXP2 were down-regulated. However, FOXP1 in Oncomine is up-regulated in cancer tissues and FOXP1 in TIME is up-regulated in normal tissues. In addition, the significant changes of FOXPs expression in transcription level between breast cancer and normal breast tissues showed in Table 1 (Oncomine database). Unpaired data analysis also showed that the mRNA expression levels of FOXP3/4 in BRCA tissues (n = 1109) were significantly higher than those in adjacent normal tissues (n = 113), and FOXP1/2 in BRCA tissues (n = 1109) were significantly lower than those in adjacent normal tissues (n = 113). (Fig. 1C, FOXP1 N: 4.851 ± 0.457 vs. T: 4.534 ± 0.804; FOXP2 N: 1.465 ± 0.481 vs. T: 0.539 ± 0.462; FOXP3 N: 1.428 ± 0.646 vs. T: 2.818 ± 1.02; FOXP4 N: 5.319 ± 0.503 vs. T: 5.819 ± 0.834, Mann–Whitney U-test, *P* < 0.001) (ns, no significance, *P < 0.05, **P < 0.01, ***P < 0.001) .

**Table 1:** The significant changes of FOXP expression in transcription level between different types of breast cancer and normal breast tissues (Oncomine database).

Type of breast cancer versus normal breast tissue	Fold change	*P* value	t test	Source
**FOXP1**				
Benign breast neoplasm	1.101	8.27E−06	5.374	Curtis breast statistics
Ductal breast carcinoma in situ epithelia	1.18	0.001	3.805	Curtis breast statistics
Invasive ductal and invasive lobular breast carcinoma	1.199	1.30E−12	7.497	Curtis breast statistics
Invasive breast carcinoma	1.216	7.96E−04	3.573	Curtis breast statistics
Invasive lobular breast carcinoma	1.208	1.49E−13	7.676	Curtis breast statistics
Mucinous breast carcinoma	1.195	7.76E−06	4.69	Curtis breast statistics
Invasive ductal breast carcinoma	1.195	5.00E−23	11.181	Curtis breast statistics
Tubular breast carcinoma	1.084	5.31E−05	3.958	Curtis breast statistics
Invasive breast carcinoma stroma	4.861	1.01E−19	18.498	Finak breast statistics
Invasive ductal breast carcinoma stroma	1.584	5.45E−04	4.541	Karnoub breast statistics
**FOXP2**				
Ductal breast carcinoma	1.273	1.73E−05	4.81	Richardson breast 2 statistics
Invasive ductal breast carcinoma	1.017	0.003	2.732	TCGA breast 2
Invasive breast carcinoma stroma	1.803	8.79E−05	5.936	Finak breast statistics
**FOXP3**				
Mucinous breast carcinoma	1.309	0.002	3.839	TCGA
Invasive breast carcinoma	1.136	0.007	2.483	TCGA
Breast carcinoma	1.148	0.002	3.384	Curtis breast statistics
Invasive breast carcinoma	1.075	0.002	3.046	Curtis breast statistics
Invasive ductal and invasive lobular breast carcinoma	1.073	2.99E−05	4.115	Curtis breast statistics
Invasive lobular breast carcinoma	1.042	0.001	2.998	Curtis breast statistics
Invasive ductal breast carcinoma	1.053	9.01E−07	4.943	Curtis breast statistics
Invasive breast carcinoma stroma	1.592	6.06E−07	10.59	Finak breast statistics
**FOXP4**				
Mucinous breast carcinoma	3.061	2.28E−05	10.005	TCGA
Invasive ductal and lobular carcinoma	1.544	2.23E−06	9.435	TCGA
Invasive ductal breast carcinoma	1.681	2.36E−24	12.939	TCGA
Invasive breast carcinoma	1.66	8.40E−13	7.866	TCGA
Mixed lobular and ductal breast carcinoma	1.224	0.002	3.233	TCGA
Invasive lobular breast carcinoma	1.343	6.07E−05	4.132	TCGA
Benign breast neoplasm	1.07	0.005	4.380	Curtis breast statistics
Medullary breast carcinoma	1.227	0.003	2.883	Curtis breast statistics
Invasive ductal breast carcinoma	1.113	1.30E−07	5.337	Curtis breast statistics
Ductal breast carcinoma	2.122	3.40E−06	5.272	Richardson breast 2 statistics
Ductal breast carcinoma in situ epithelia	1.46	0.004	3.282	Ma breast 4 statistics
Invasive breast carcinoma stroma	1.308	1.80E−10	9.534	Finak breast statistics
Invasive ductal breast carcinoma	1.041	2.28E−08	5.534	TCGA breast 2

### Relationships between FOXPs mRNA levels and clinical characteristics of BRCA patients

To evaluate the association between the mRNA expression of FOXPs and clinical pathological characteristics of BRCA samples, we performed Mann–Whitney U-test analysis. As shown in Fig. 1D, FOXP2,3,4 mRNA expression were significantly higher in patients (< 60) than in patients (> = 60) (p.adj < 0.001, p.adj = 0.006, p.adj < 0.001), however the result was the opposite in FOXP1(P = 0.009). No statistically significant correlation were found between the expression levels of FOXPs in Pathologic stage (p.adj > 0.05) (Fig. 1E). We used the Bonferroni method to correct the multiple hypothesis test (Dunn's test) of significance level. The results of Race showed that FOXP1 expression was lower in black or African American than in Asian, and the difference was statistically significant (p.adj = 0.001); FOXP2 expression was lower in black or African American than in Asian, and the difference was not statistically significant (p.adj = 1); No statistically significant correlation were found between the expression levels of FOXPs and Race (p.adj > 0.05); FOXP4 expression was lower in black or African American than Asian, and the difference was not statistically significant (p.adj = 1) (Fig. 1F). The results of Histological type showed that FOXP1,2 mRNA expression were significantly higher in Infiltrating Lobular Carcinoma than Infiltrating Ductal Carcinoma (p.adj < 0.001), however no statistically significant correlation were found between FOXP3,4 and Histological type (p.adj > 0.05) (Fig. 1G). (ns, no significance, *P < 0.05, **P < 0.01, ***P < 0.001).

We used immunohistochemistry (IHC) to detect the protein expression of FOXPs in BRCA and its paired adjacent tissues. The results showed that FOXPs protein express in the nucleus. FOXPs protein express in the nucleus. We found that the protein levels of FOXP3 and FOXP4 were higher in BRCA tissues than in the adjacent tissues, however the result was the opposite in FOXP1 and FOXP2 (Fig. 1H).

### The prognostic value of FOXPs in BRCA patients

**Figure 2:**
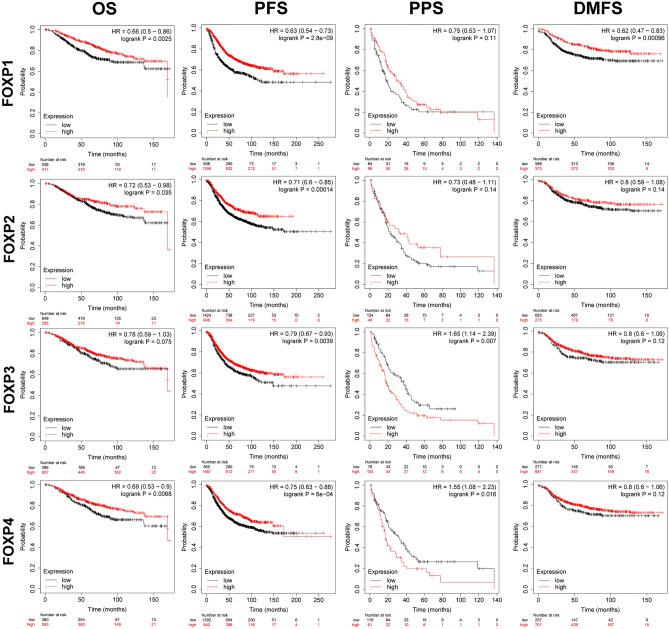
The prognostic value of mRNA Level of FOXPs in BRCA patients (Kaplan–Meier plotter). Overall survival (OS), progression-free survival (PFS), post-progression survival (PPS) and distant metastasis-free survival (DMFS).

To evaluate the value of FOXPs at different transcription levels in the progression of BRCA, we evaluated the correlation between FOXPs at different transcription levels and clinical outcome using Kaplan–Meier plotter analysis. The OS curve is shown in Fig. 2. BRCA patients with high transcription levels of FOXP1/2/4 were significantly associated with longer OS. In contrast, BRCA patients with transcription levels of FOXP3 was not statistically related with OS. In addition, the studies showed that BRCA with high expression of FOXP1/2/3/4 was significantly associated with longer PFS (Fig. 2). While transcription levels of FOXP1/2 were not related to PPS in BRCA patients. In contrast, BRCA patients with low transcription levels of FOXP3/4 were significantly associated with longer PPS (Fig. 2). The value of FOXPs at different transcription levels in DMFS of BRCA patients was also evaluated. Only BRCA patients with high mRNA expression of FOXP1 were significantly related with longer DMFS (Fig. 2).

### Co-expression of FOXPs in BRCA patients

**Figure 3:**
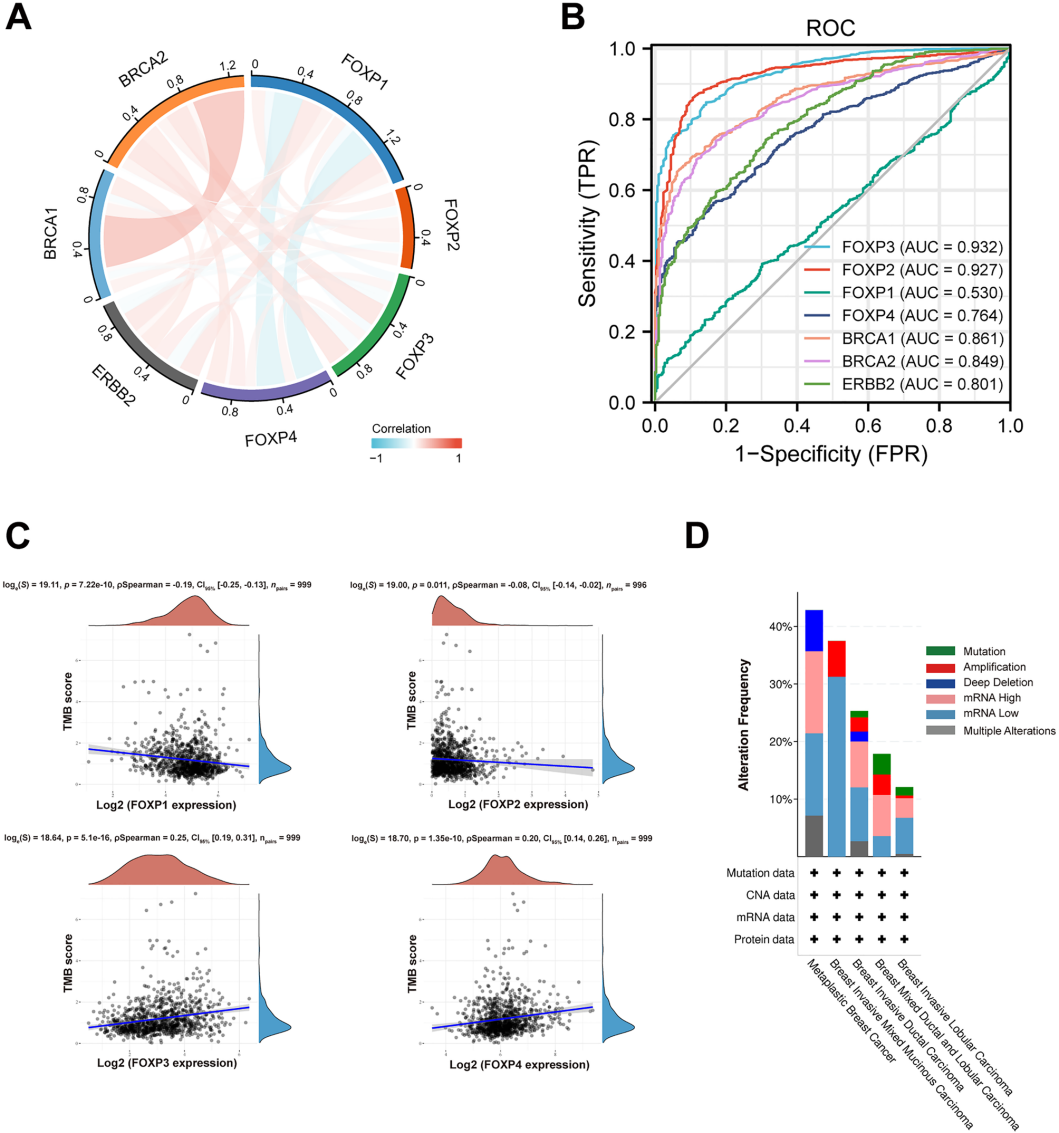
Relevance of different FOXPs and important genes, diagnostic value, TMB score and mutation analysis in BRCA. (**A**) Correction between different FOXPs and Important Genes in BRCA. (**B**) ROC curve analysis was performed to evaluate the diagnostic power of the FOXPs and Important Genes. (**C**) Correlation analysis of FOXPs expression and TMB in BRCA. The horizontal axis in the figure represents the expression distribution of the gene, and the ordinate is the expression distribution of the TMB score. The density curve on the right represents the distribution trend of the TMB score; The upper density curve represents the distribution trend of the gene; The top side: the value represents the correlation p value, correlation coefficient and correlation calculation method. (**D**) Summary of alterations in different expressed FOXPs and immune checkpoint in BRCA (cBioPortal).

Evaluation of the mutual exclusion between the four FOXPs genes in the TCGA BRCA cohort showed that there were co-expressed relationships between FOXP1 and FOXP2/FOXP3/FOXP4/ERBB2/BRCA1, FOXP2 and FOXP3/BRCA2, FOXP3 and FOXP4/ERBB2/BRCA2, FOXP4 and ERBB2/BRCA1/BRCA2, ERBB2 and BRCA1/BRCA2, BRCA1 and BRCA2 (*P* < 0.05). However, FOXP1 with BRCA2, FOXP2 with FOXP4/ERBB2/BRCA1 and FOXP3 with BRCA1 didn’t have co-expressed relationships (*P* > 0.05) (Fig. 3A).

### Differential RNA-Seq levels of FOXPs as prospective biomarker to distinguish BRCA samples from normal samples and the TMB score of FOXPs in BRCA

To investigate the value for FOXPs to distinguish Breast Invasive Carcinoma samples from normal smples, we performed a ROC curve analysis using ERBB2, BRCA1 and BRCA2 as controls. As showed in (Fig. 3B) (Table S1), the ROC curve analysis showed FOXP1 (AUC: 0.530, accuracy: 0.862), FOXP2 (AUC: 0.927, accuracy: 0.968), FOXP3 (AUC: 0.932, accuracy: 0.957), FOXP4 (AUC: 0.764, accuracy: 0.928), ERBB2 (AUC: 0.801, accuracy: 0.900), BRCA1 (AUC: 0.861, accuracy: 0.973), BRCA2 (AUC: 0.849, accuracy: 0.957). These fingerings indicated that FOXP2 and FOXP3 could be a promising biomarker to differentiate Breast Invasive Carcinoma tissues from normal tissues. In addition, we made a TMB scoring model (Fig. 3C).

### Genetic alteration of FOXPs and immune checkpoint in BRCA patients

**Figure 4:**
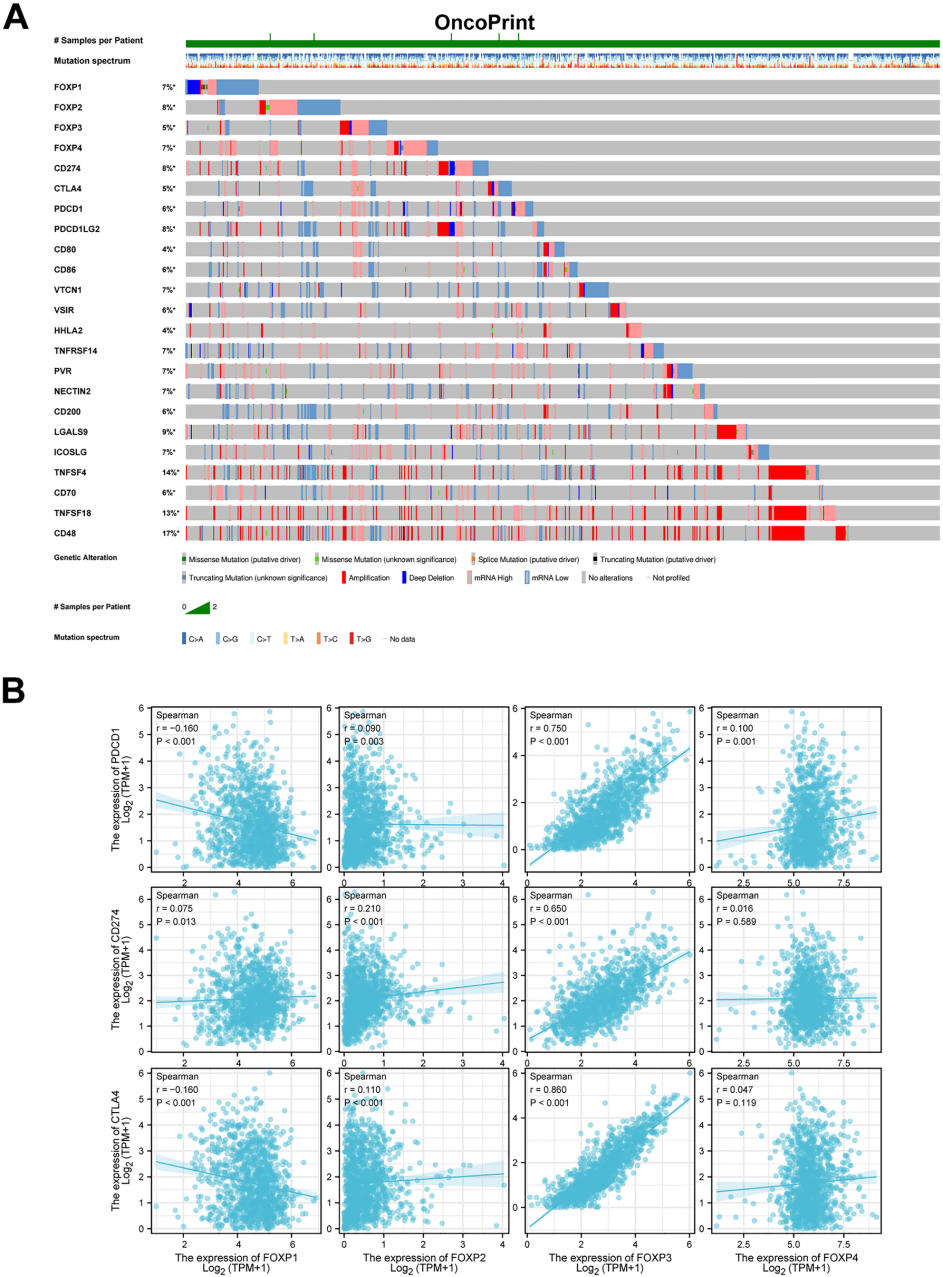
FOXPs and immune checkpoints mutation analysis and the relationship between the expression of FOXPs and immune checkpoints genes (PDCD1, CD274, CTLA4) in BRCA. (**A**) Landscape of FOXPs and immune checkpoint alteration in Breast Invasive Carcinoma (cBioPortal). (**B**) FOXP3 has a significant positive correlation with PDCD1, CD274, CTLA4, but FOXP1, 2, 4 didn’t.

We used the cBioPortal online tool to analyze changes and correlations of FOXPs in Breast Invasive Carcinoma. Among 1108 Breast Invasive Carcinoma patients, FOXPs were changed in 258 samples (23.28%) (Fig. 3D). The genomic investigation revealed that FOXPs was actually involved in the alteration of immune checkpoints in Breast Invasive Carcinoma. The general landscape of FOXPs and immune checkpoint alteration in Breast Invasive Carcinoma was compactly visualized, including structural variant, mRNA, amplification, deep deletion, truncating, splice and missense mutations (Fig. 4A). The detailed relationship between FOXPs and each representative immune checkpoint was individually presented as indicated in Table 2. Of note, the FOXP3 alteration showed a statistically significant co-occurrence rather than mutual exclusivity with extensive immune checkpoints, such as CTLA4, CD48, PDCD1, CD70, PDCD1LG2, CD86, CD80, CD274, ICOSLG. These findings strongly indicate that FOXP3 is a potential coregulator of immune checkpoints in Breast Invasive Carcinoma.

**Table 2:** Mutual-exclusivity analysis between FOXPs and multiple-immune checkpoints in BRCA.

A	B	Neither	A Not B	B Not A	Both	Log2 odds ratio	*p* value	q-Value	Tendency	Significant
FOXP1	PVR	651	41	34	12	2.486	< 0.001	< 0.001	Co-occurrence	Yes
FOXP1	NECTIN2	645	41	40	12	2.239	< 0.001	< 0.001	Co-occurrence	Yes
FOXP1	CD70	650	45	35	8	1.723	0.008	0.027	Co-occurrence	Yes
FOXP1	TNFSF4	604	41	81	12	1.126	0.025	0.064	Co-occurrence	No
FOXP1	VTCN1	637	45	48	8	1.238	0.039	0.092	Co-occurrence	No
FOXP1	CD86	646	46	39	7	1.334	0.039	0.092	Co-occurrence	No
FOXP1	HHLA2	661	48	24	5	1.521	0.05	0.108	Co-occurrence	No
FOXP1	TNFRSF14	643	46	42	7	1.22	0.053	0.11	Co-occurrence	No
FOXP1	CD200	648	47	37	6	1.161	0.079	0.15	Co-occurrence	No
FOXP1	ICOSLG	633	46	52	7	0.889	0.12	0.205	Co-occurrence	No
FOXP1	PDCD1	642	47	43	6	0.931	0.13	0.218	Co-occurrence	No
FOXP1	CD48	576	41	109	12	0.629	0.14	0.229	Co-occurrence	No
FOXP1	CD80	658	49	27	4	0.992	0.176	0.272	Co-occurrence	No
FOXP1	PDCD1LG2	636	47	49	6	0.729	0.194	0.286	Co-occurrence	No
FOXP1	VSIR	646	48	39	5	0.787	0.201	0.293	Co-occurrence	No
FOXP1	TNFSF18	604	45	81	8	0.407	0.301	0.393	Co-occurrence	No
FOXP1	CD274	631	48	54	5	0.284	0.42	0.495	Co-occurrence	No
FOXP1	LGALS9	629	48	56	5	0.227	0.45	0.513	Co-occurrence	No
FOXP1	CTLA4	648	51	37	2	− 0.542	0.457	0.517	Mutual exclusivity	No
FOXP2	HHLA2	658	51	21	8	2.297	0.001	0.006	Co-occurrence	Yes
FOXP2	VTCN1	633	49	46	10	1.49	0.009	0.029	Co-occurrence	Yes
FOXP2	CD200	644	51	35	8	1.529	0.016	0.045	Co-occurrence	Yes
FOXP2	NECTIN2	636	50	43	9	1.413	0.017	0.047	Co-occurrence	Yes
FOXP2	CTLA4	647	52	32	7	1.445	0.029	0.072	Co-occurrence	No
FOXP2	CD70	643	52	36	7	1.266	0.047	0.104	Co-occurrence	No
FOXP2	VSIR	642	52	37	7	1.224	0.053	0.11	Co-occurrence	No
FOXP2	PDCD1LG2	632	51	47	8	1.077	0.062	0.126	Co-occurrence	No
FOXP2	CD86	640	52	39	7	1.143	0.065	0.131	Co-occurrence	No
FOXP2	TNFRSF14	637	52	42	7	1.03	0.086	0.159	Co-occurrence	No
FOXP2	CD80	653	54	26	5	1.218	0.093	0.167	Co-occurrence	No
FOXP2	CD48	571	46	108	13	0.579	0.15	0.242	Co-occurrence	No
FOXP2	CD274	627	52	52	7	0.699	0.182	0.275	Co-occurrence	No
FOXP2	ICOSLG	627	52	52	7	0.699	0.182	0.275	Co-occurrence	No
FOXP2	TNFSF4	596	49	83	10	0.551	0.195	0.286	Co-occurrence	No
FOXP2	PVR	638	54	41	5	0.527	0.303	0.393	Co-occurrence	No
FOXP2	LGALS9	624	53	55	6	0.361	0.36	0.444	Co-occurrence	No
FOXP2	PDCD1	633	56	46	3	− 0.44	0.436	0.504	Mutual exclusivity	No
FOXP2	TNFSF18	597	52	82	7	− 0.029	0.581	0.6	Mutual exclusivity	No
FOXP3	CTLA4	676	23	29	10	> 3	< 0.001	< 0.001	Co-occurrence	Yes
FOXP3	CD48	600	17	105	16	2.427	< 0.001	< 0.001	Co-occurrence	Yes
FOXP3	PDCD1	666	23	39	10	2.892	< 0.001	< 0.001	Co-occurrence	Yes
FOXP3	CD70	671	24	34	9	2.888	< 0.001	< 0.001	Co-occurrence	Yes
FOXP3	PDCD1LG2	660	23	45	10	2.673	< 0.001	< 0.001	Co-occurrence	Yes
FOXP3	CD86	668	24	37	9	2.759	< 0.001	< 0.001	Co-occurrence	Yes
FOXP3	CD80	681	26	24	7	2.933	< 0.001	0.001	Co-occurrence	Yes
FOXP3	CD274	655	24	50	9	2.296	< 0.001	0.004	Co-occurrence	Yes
FOXP3	ICOSLG	655	24	50	9	2.296	< 0.001	0.004	Co-occurrence	Yes
FOXP3	CD200	667	28	38	5	1.648	0.037	0.089	Co-occurrence	No
FOXP3	LGALS9	650	27	55	6	1.393	0.047	0.104	Co-occurrence	No
FOXP3	VTCN1	654	28	51	5	1.195	0.097	0.172	Co-occurrence	No
FOXP3	TNFRSF14	660	29	45	4	1.016	0.169	0.263	Co-occurrence	No
FOXP3	TNFSF4	618	27	87	6	0.659	0.227	0.32	Co-occurrence	No
FOXP3	PVR	662	30	43	3	0.622	0.339	0.425	Co-occurrence	No
FOXP3	TNFSF18	621	28	84	5	0.401	0.366	0.449	Co-occurrence	No
FOXP3	NECTIN2	656	30	49	3	0.421	0.415	0.494	Co-occurrence	No
FOXP3	VSIR	663	31	42	2	0.026	0.599	0.609	Co-occurrence	No
FOXP3	HHLA2	677	32	28	1	− 0.404	0.624	0.627	Mutual exclusivity	No
FOXP4	NECTIN2	643	43	38	14	2.462	< 0.001	< 0.001	Co-occurrence	Yes
FOXP4	ICOSLG	634	45	47	12	1.847	< 0.001	0.005	Co-occurrence	Yes
FOXP4	PDCD1LG2	636	47	45	10	1.588	0.006	0.022	Co-occurrence	Yes
FOXP4	PDCD1	640	49	41	8	1.35	0.028	0.07	Co-occurrence	No
FOXP4	TNFRSF14	640	49	41	8	1.35	0.028	0.07	Co-occurrence	No
FOXP4	CD70	645	50	36	7	1.327	0.04	0.093	Co-occurrence	No
FOXP4	CD48	574	43	107	14	0.805	0.066	0.131	Co-occurrence	No
FOXP4	VSIR	638	56	43	1	− 1.916	0.128	0.216	Mutual exclusivity	No
FOXP4	TNFSF18	602	47	79	10	0.697	0.134	0.222	Co-occurrence	No
FOXP4	CD200	643	52	38	5	0.702	0.232	0.324	Co-occurrence	No
FOXP4	CD80	651	56	30	1	− 1.368	0.291	0.388	Mutual exclusivity	No
FOXP4	CD274	628	51	53	6	0.479	0.3	0.393	Co-occurrence	No
FOXP4	CTLA4	644	55	37	2	− 0.66	0.405	0.491	Mutual exclusivity	No
FOXP4	TNFSF4	596	49	85	8	0.195	0.431	0.502	Co-occurrence	No
FOXP4	VTCN1	630	52	51	5	0.248	0.439	0.505	Co-occurrence	No
FOXP4	CD86	638	54	43	3	− 0.279	0.517	0.558	Mutual exclusivity	No
FOXP4	PVR	638	54	43	3	− 0.279	0.517	0.558	Mutual exclusivity	No
FOXP4	LGALS9	625	52	56	5	0.102	0.518	0.558	Co-occurrence	No
FOXP4	HHLA2	654	55	27	2	− 0.183	0.609	0.616	Mutual exclusivity	No

### The relationship between the expression of FOXPs with PDCD1, CD274, CTLA4 in BRCA

The two-gene correlation map was realized by the R software package “ggstatsplot”, and data from TCGA.We found that FOXP3 had a significant positive correlation with PDCD1, CD274, CTLA4 (Fig. 4B). However, FOXP1/2/4 was weakly associated with PDCD1, CD274, CTLA4 (Fig. 4B).

### The relationship between FOXPs expression levels and immune infiltration levels in BRCA

**Figure 5:**
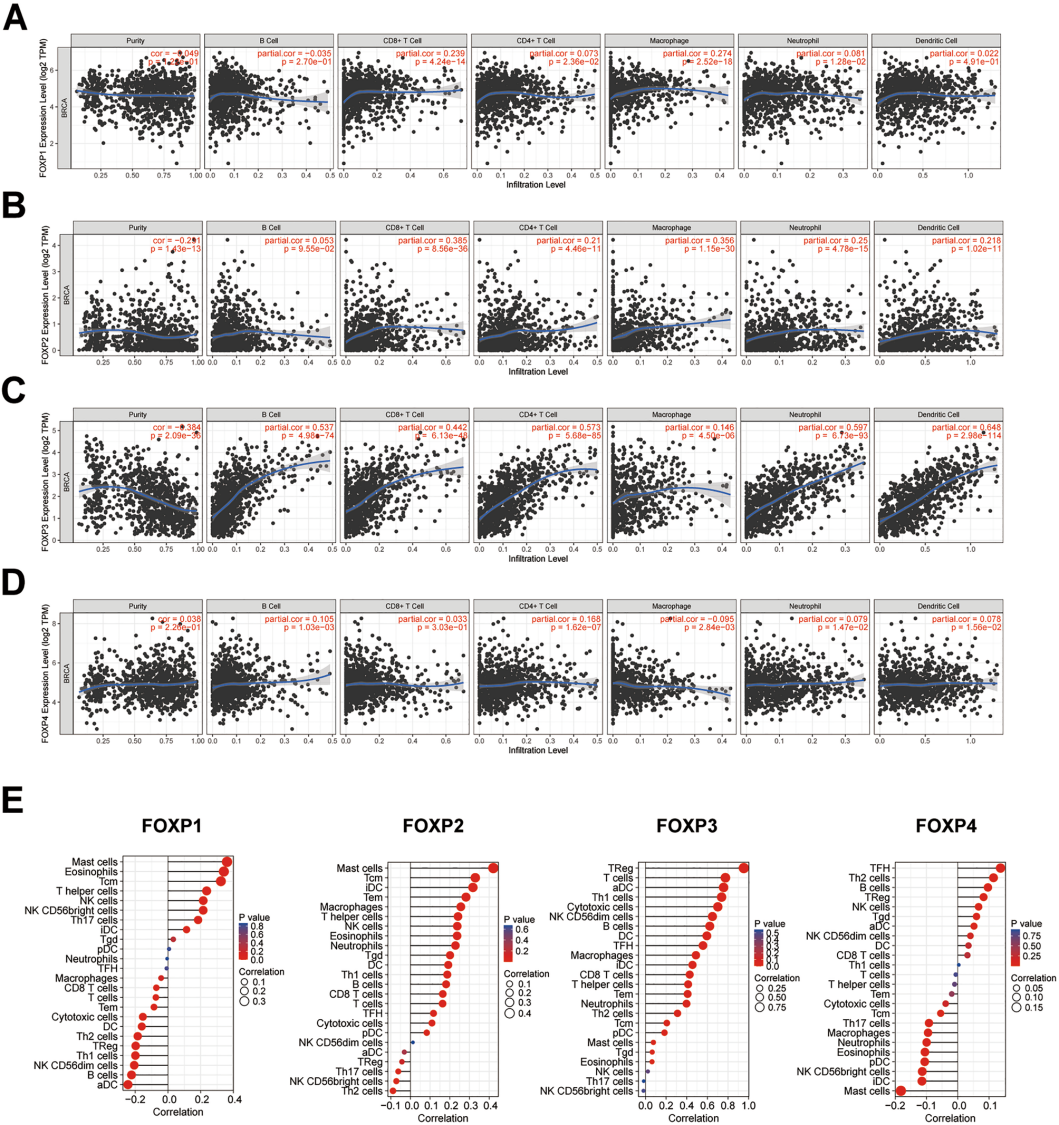
The relationship between FOXPs expression levels and immune infiltration levels in BRCA. The correlation between the abundance of immune cell and the expression of (**A**) FOXP1, (**B**) FOXP2, (**C**) FOXP3, (**D**) FOXP4 in BRCA (TIMER). (**E**) Lollipop chart about correlation between 24 immune cell and the expression of FOXPs in BRCA.

TIMER online analysis tool is used to evaluate the relationship between the transcription level of FOXPs and the level of immune infiltration in BRCA. It was found that FOXPs are involved in inflammatory response and immune cell infiltration. The analysis results are shown in Fig. 5. FOXP1 expressions was positively correlated with the infiltration of CD4+ T cells, CD8+ T cells, neutrophils and macrophages (Fig. 5A). FOXP2 expressions was positively correlated with CD4+ T cells, CD4+ T cells, neutrophils, macrophages and dendritic cells (Fig. 5B). FOXP3 expression was positively correlated with infiltration of B cells, CD4+ T cells, CD4+ T cells, neutrophils, macrophages and dendritic cells (Fig. 5C). FOXP4 expressions was positively correlated with the infiltration of B cells, CD4+ T cells, neutrophils and macrophages, while it was negatively correlated with infiltration of Fig. 5D. These studies indicated that the level of FOXPs expression was associated to the level of immune infiltration in BRCA. At the same time, we made four Lollipop charts 24 immune cells associated with FOXPs expression in BRCA to have a more intuitive understanding of the immune infiltration (Fig. 5E). We can see that there were most immune cell infiltration in FOXP2 and FOXP3.

### PPI and neighbor gene network of FOXPs in BRCA patients

**Figure 6:**
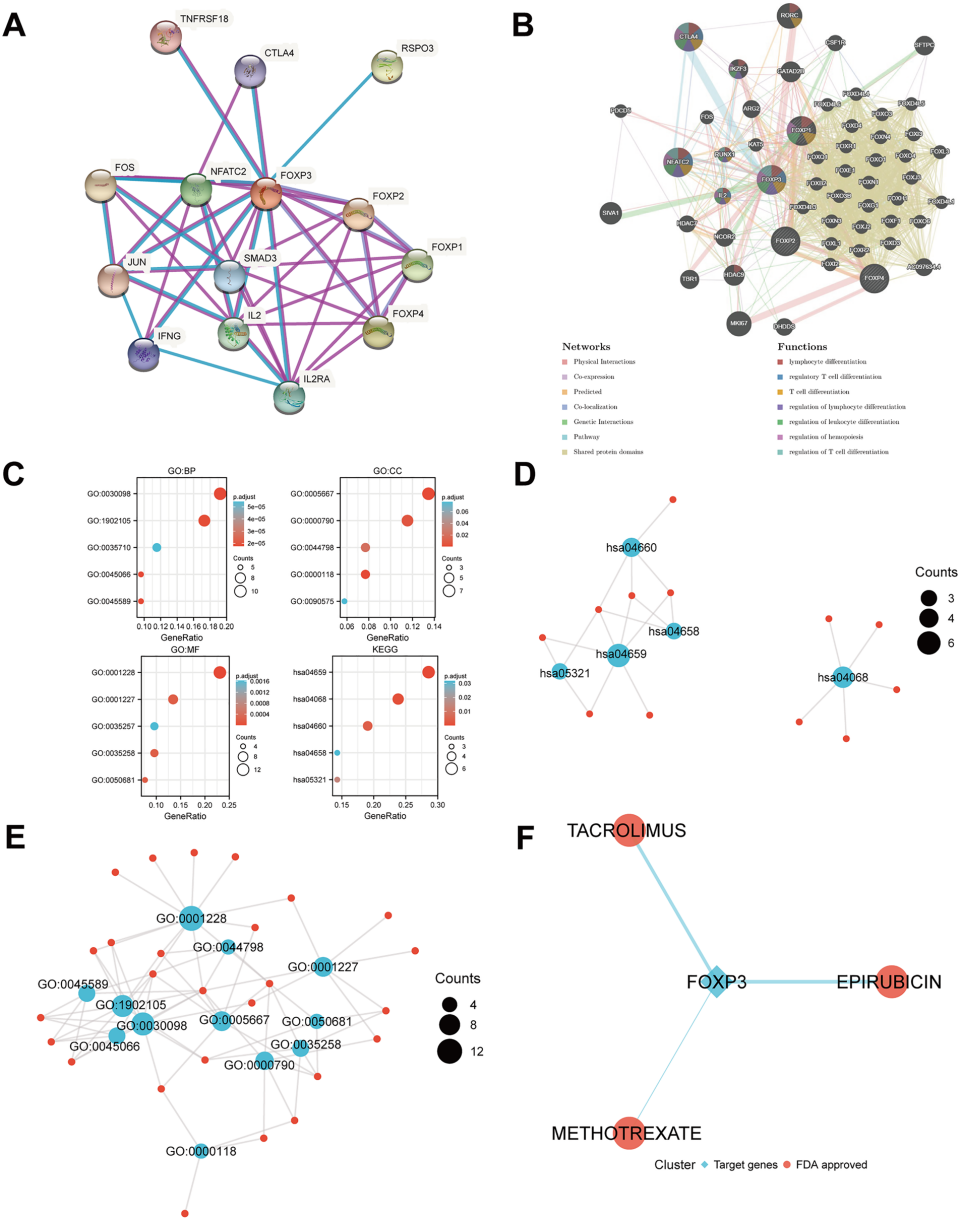
PPI network, neighbor gene network, interaction analyses of FOXPs, the functions of FOXPs, genes significantly associated with FOXPs alterations in BRCA and drug–gene interaction. (**A**) Protein–protein interaction network of different expressed FOXPs (STRING). (**B**) Gene–gene interaction network of different expressed FOXPs (GeneMANIA). (**C**) GO enrichment analysis predicted the functional roles of target host genes based on three aspects, including biological processes, cellular components, and molecular functions. The functions of FOXPs and genes significantly associated with FOXPs alterations were predicted by the analysis of KEGG. (**D**,**E**) Network of GO and KEGG enriched terms. (**F**) FOXP3 has significant correlations with drug-gene interaction.

We performed a PPI network analysis of FOXPs at different transcription levels using STRING to study the potential interactions between them. As shown in (Fig. 6A), the PPI network diagram contains four FOXPs proteins and 10 proteins that are closely related to FOXPs.

A GGI network of four FOXPs was constructed, and their functions were analyzed using the GeneMANIA database (Fig. 6B). The four central nodes of FOXPs are surrounded by 50 nodes representing genes that are strongly associated with FOXPs in shared protein domains, physical interactions, colocalization, co-expression, prediction, genetic interactions, and pathway. The top 50 genes most associated with FOXPs are NFATC2, CTLA4, RORC, MKI67, SIVA1, HDAC9, GATAD2B, IKZF3, SFTPC, TBR1 and so on. Among them, NFATC2 is related to FOXP1/2/3/4 in terms of physical interaction and genetic interaction. NFATC2 and CTLA4 have a pathway relationship with FOXP3. CTLA4 is related to FOXP3 in terms of co-localization. However, RORC, MKI67, SIVA1, HDAC9, GATAD2B, IKZF3, SFTPC, TBR1 and FOXP1/2/3/4 all have physical interaction. Further functional analysis showed that these genes indicated the greatest correlation with lymphocyte differentiation (FDR = 1.54E-4). In addition, these genes were correlated with regulatory T cell differentiation, T cell differentiation, regulation of lymphocyte differentiation, regulation of leukocyte differentiation, regulation of hemopoiesis and regulation of T cell differentiation.

### Functional enrichment analysis of FOXPs in BRCA patients

In this study, we used “ClusterProfiler” R package to perform functional annotation and pathway enrichment analysis of FOXPs from 50 nodes representing genes. The first 15 items of GO enrichment (Table S2) are mainly distributed in the biological process (5 items) (Fig. 6C), the cell component (5 items) (Fig. 6C) and the molecular function (5 items) (Fig. 6C). Three of the first five projects are in the T cell function, which are regulation of regulatory T cell differentiation, regulatory T cell differentiation, lymphocyte differentiation, and the other two are regulation of leukocyte differentiation and DNA-binding transcription activator activity, RNA polymerase II-specific.

The first 5 KEGG pathways of FOXPs are illustrated in Fig. 6C (Table S2). Among them, Th17 cell differentiation, FoxO signaling pathway, T cell receptor signaling pathway, Inflammatory bowel disease and Th1 and Th2 cell differentiation are significantly associated with the occurrence and development of various tumors and are also involved in the tumorigenesis of BRCA.

At the same time, we made more intuitive GO network map (Fig. 6D) and KEGG network map to show the connection between pathways (Fig. 6E).

### Drug–gene interactions

The result showed expressions of FOXP3 had significant correlations with drug-gene interaction. A total of 3 drugs were explored using DGIDB that might have potential to treat affected patient, Epirubicin, Tacrolimus and Methotrexate (Fig. 6F).

## Discussion

The previous studies have revealed that the dysregulation of FOXPs is significantly related to the carcinogenesis and progression of many tumors^13–15,31,32^. In this study, database analysis showed that the transcription levels of FOXPs in many human tumors were frequently altered. Although the role of FOXPS in the carcinogenesis, development and prognosis of some cancers has been partially elucidated, there have been no further bioinformatics studys of different FOXPs expression and function in breast cancer. This study is the first time to investigate the mRNA expression, gene variation, molecular mechanism, and biological function of different FOXP factors in breast cancer and its influence on the prognosis and immune infiltration in patients with breast cancer through bioinformatics analysis.

Among all members of FOXPs, FOXP1 gene has been mapped to chromosome 3p14.1, a region that it has been detected widespread loss of heterozygosity in breast cancer^33^, particularly of breast cancer with BRCA2 mutations^34^. Fox SB et al.^35^ found that nuclear protein expression of FOXP1 was significantly positively related to estrogen receptor status but not associated with tumor size, age, lymph node status, or grade. In addition, FOXP1 co-expression with estrogen receptor significantly improved relapse-free survival,it suggests FOXP1 may function as a tumour suppressor in breast cancer. FOXP1 could modulate cell proliferation in breast cancer cells and improve 5-year recurrence-free survival of patients with tamoxifen-treated breast cancer from Shigekawa T et al.’s study^36^. Similarly,a report confirmed that the increased FOXP1 protein expression could predict a good effect to tamoxifen in breast carcinoma patients^37^. A systematic review and meta-analysis revealed that decreased FOXP1 protein expression was significantly associated with an unfavorable relapse-free survival (RFS) in breast cancer patients^38^. In this study, database analysis showed that the transcription levels of FOXP1 in human breast cancer were lower than in normal tissues, and immunohistochemical staining from our breast carcinoma specimen also demonstrated this result. But the expression of FOXP1 in patients with breast cancer were not associated with the tumor stage. In addition, Our research was similar to Jian X et al. ’s study^38^, low FOXP1 transcription levels were associated with poor OS, PFS and DMFS in patients with breast cancer.

Recently, some roles of FOXP2 have been verified in cancer development as a tumor suppressor, though its mutations could cause language disorders. Also, Cuiffo et al.^39^ found that downregulation of FOXP2 strengthened tumor initiation in breast carcinoma and promoted cancer stem cell metastasis. Furthermore, Chen et al.^40^ reported that the transcription level of FOXP2 in breast cancer tissue was also markedly lower than in normal breast tissue and these patients also had poor RFS rate. Similarly, In this study, database analysis found that the transcription levels of FOXP2 in human breast cancer were lower than in normal tissues, and similar result also was found in our specimen by immunohistochemical staining. But the expression of FOXP2 in breast cancer patients has nothing to do with the tumor histological type. It was also found that the low transcription levels of FOXP2 in breast cancer patients correlated with poor OS, PFS.

FOXP3 plays an important role in regulating Treg cells development and functions for immune response against cancer^41^. FOXP3 also inhibited growth and induced the cell death of a breast cancer cell line MCF-7^42^. In addition, Some studies have demonstrated that FOXP3 is an important tumor suppressor of oncogenes in breast cancer with good prognosis^42–44^. The database analysis and IHC in this study testified that the expression levels of FOXP3 in human breast cancer were higher than in normal tissues, and its expression levels were not related to the tumor stage, histological type and race. The survival analysis found that high transcription levels of FOXP3 in breast cancer patients resulted in worse PPS and had better PFS.

Previous studies indicated that FOXP4 had dual biologic function as a tumor suppressor in patients with kidney cancer^45^, or as an oncogene in in patients with hepatocellular carcinoma^46^. In present study, the database analysis and IHC revealed that the expression levels of FOXP4 in human breast cancer were higher than in normal tissues, which was consistent with Ma et al.^47^ results and its expression levels were not related to the tumor stage, histological type and pathologic stage. In addition, Ma et al.^47^ results showed that high expression of FOXP4 predicted a poor OS in breast canccer patients, contrastly, in this study, low FOXP4 expression levels were associated with poor OS and PFS in patients with breast cancer, interestingly, except PPS.

Furthermore, a high gene alteration rate of FOXPs was foundin breast cancer patients, and there were difference gene alteration rate in different histological type of breast cancer. Moreover, a mutually exclusive or co-occurring connection between FOXPs or between FOXPs and BRCA or ERBB2 was different, suggesting that these gene play an different role in development of breast cancer.

Previous reports showed that FOXP2 and FOXP3 might be a potential biomarker for breast cancer^48,49^. Consistantly, in this study, we performed ROC curve analysis. Our results showed that FOXP2 or FOXP3 had a significantly high AUC value in the detection of breast cancer, with 96.8% or 95.7% in accuracy respectively. On the basis of these findings, we conclude that FOXP2 and FOXP3 might act as a potential diagnostic biomarker to differentiate breast cancer from normal normal tissues.

Accumulating evidence demonstrated that FOXPs proteins play important roles in the regulation of immune function^50,51^. In this work, genomic analysis revealed that FOXPs was closely related to the alteration of extensive immune checkpoints in breast invasive carcinoma. Importantly, the connection between FOXP3 alteration with extensive immune checkpoints was co-occurrence but not mutual exclusivity.

Additionally, we found that FOXP3 had a significant positive correlation with PDCD1, CD274, CTLA4 and TMB in breast cancer. This study yet demonstrated that FOXPs were involved in inflammatory response and immune cell infiltration, of note, FOXP3 expression showed a statistically significant correlation with infiltration of B cells, CD4+ T cells, CD4+ T cells, neutrophils, macrophages and dendritic cells. Consistantly, West et al.^52^ reported that the breast cancer patients with FOXP3+ TILs had better survival. These findings strongly indicate that FOXP3 is a potential regulator of immune in breast invasive carcinoma.

Previous studies showed that FOXPs were associated with a great deal of genes or proteins, such as TNF receptor family-related gene (GITR)^53^, cytooxic T lymphocyte associated antigen 4 (CTLA-4) and CD25^54^, TGF-β^55^, nuclear factor of activated T cells (NFAT)^55^, Runt-related transcription factor 1 (RUNX1)^56^. In this study, we also analysed relation of FOXPs and its neighboring genes or proteins, and found that the main 50 genes were associated with FOXPs. Further analysis showed that the functions of these proteins are mainly related to lymphocyte differentiation and regulation of lymphocyte function.

Another significant result of this study revealed that FOXP3 expression predicted the breast cancer cells’ response to anticancer drugs, whereas FOXP1, FOXP2 and FOXP4 did not predict. Consistantly, Ladoire et al.’s^47^ report showed that FOXP3 expression in breast cancer was independently related to improved OS in patients treated with anthracycline-based adjuvant chemotherapy.

## Conclusions

In conclusion, Our results suggested that BRCA patients with high transcription levels of FOXP1/2/4 had better prognosis and FOXPs was closely related to the alteration of extensive immune checkpoints in breast invasive carcinoma. FOXP3 expression showed a statistically significant correlation with infiltration of B cells, CD4+ T cells, CD4+ T cells, neutrophils, macrophages and dendritic cells and predicted the breast cancer cellsʼ s response to anticancer drugs, the main 50 genes were involved in FOXPs, our study suggested that FOXPs could serve as a biomarker for tumor detection, therapeutic design and prognosis.

## Supplementary Information

Supplementary Table S1.

Supplementary Table S2.

## Data Availability

These data are drawn from the public domain. The datasets used and/or analysed during the current study available from the corresponding author on reasonable request.

## Abbreviations

FOXP: Forkhead box P
BRCA: Breast cancer
PPI network: Protein protein interaction network
